# Case report: A novel compound heterozygous variant in the *TNXB* gene causes single kidney agenesis and vesicoureteral reflux

**DOI:** 10.3389/fendo.2024.1322395

**Published:** 2024-02-02

**Authors:** Lei Liang, Haotian Wu, Haixia Meng, Lin Fu, Jianrong Zhao

**Affiliations:** ^1^ Center for Prenatal Diagnosis and Medical Genetics, Affiliated Hospital of Inner Mongolia Medical University, Hohhot, China; ^2^ School of Public Health, Inner Mongolia Medical University, Hohhot, China; ^3^ Department of Ultrasound, Affiliated Hospital of Inner Mongolia Medical University, Hohhot, China; ^4^ Department of Nephrology, Affiliated Hospital of Inner Mongolia Medical University, Hohhot, China

**Keywords:** vesicoureteral reflux, TNXB, compound heterozygous variant, single kidney, molecular dynamics

## Abstract

Primary vesicoureteral reflux (VUR) is the prevailing congenital anomaly of the kidneys and urinary tract, posing a significant risk for pyelonephritis scarring and chronic renal insufficiency in pediatric patients. Nevertheless, the precise genetic etiology of VUR remains enigmatic. In this current investigation, we conducted whole-exome sequencing on a child exhibiting single kidney, devoid of any familial VUR background, along with both biological parents. Two missense variants (NM_019105.8: exon11: c.4111G>A and NM_019105.8: exon2: c.31A>T) in the *TNXB* gene were identified through whole-exome sequencing of the child. These variants were found to be inherited from the child’s parents, with each parent carrying one of the variants. Molecular dynamics simulations were conducted to assess the impact of these variants on the tenascin XB proteins encoded by them, revealing varying degrees of impairment. Based on our findings, it is suggested that the *TNXB* compound heterozygous variant, consisting of c.4111G>A and c.31A>T, may be the underlying cause of right renal agenesis and left hydronephrosis in afflicted child. This discovery broadens the genetic range of the *TNXB* gene and establishes a genetic foundation for disease-specific preimplantation genetic diagnosis (PGD) in prospective pregnancies involving the parents of this afflicted child.

## Lay summary

Primary vesicoureteral reflux (VUR) is the most prevalent congenital malformation of the kidneys and urinary tract. However, the occurrence of this disease in combination with a single kidney is exceptionally rare. In our study, we conducted whole-exome sequencing of a child with a single kidney and no familial history of VUR, as well as both of his parents. Through this analysis, we identified two missense variants (c.4111G>A and c.31A>T) in the *TNXB* gene, which were inherited from the patient’s parents. To further understand the impact of these variants, we utilized molecular dynamics simulation to assess the extent of damage caused to the encoded tenascin XB protein by each variant. Our findings suggest that the *TNXB* compound heterozygous variant is responsible for the patient’s right renal hypoplasia and left hydronephrosis.

## 1 Introduction

Primary vesicoureteral reflux (VUR) refers to the abnormal retrograde flow of urine from the bladder into the upper urinary tract, which occurs as a result of an anomaly at the ureter bladder junction (1, 2). This condition is recognized as the prevailing congenital anomaly of the kidney and urinary tract (CAKUT) among children and is considered the most significant risk factor for pyelonephritis (2). The estimated prevalence of VUR in children ranges from 1-2% (3), although this figure may be an underestimation due to the presence of phenotypic heterogeneity and the absence of non-invasive diagnostic methods. Failure to treat VUR is identified as the most significant risk factor for the development of renal parenchymal scarring (RPS) in the pediatric population (1, 2). Reflux nephropathy, which is characterized by RPS caused by VUR, is the primary cause of end-stage renal disease necessitating dialysis and kidney transplantation in children. Despite the incomplete understanding of the pathogenesis of VUR, it is hypothesized to arise from a developmental anomaly resulting from impaired reciprocal signaling interactions between the posterior renal mesenchyme and the ureteric buds during the process of kidney development (4). The human uroepithelial lining of the ureteropelvic junction expresses *TNXB*, suggesting that *TNXB* may be important in generating the tensile force that closes the ureteropelvic junction during urination (5). In our recent study, we have discovered two previously unreported compound heterozygous variants, namely c.4111G>A and c.31A>T, in the tenascin protein XB gene (*TNXB*) of a child presenting with right-sided renal agenesis and left-sided hydronephrosis. The tenascin proteins belong to a class of extracellular matrix proteins that exhibit a relatively similar structure, characterized by an N-terminal assembly and a C-terminal fibrinogen-like structural domain, as well as epidermal growth factor (EGF)-like repeat sequences and fibronectin III structural domains (6–8). These fibronectin domains play a crucial role in regulating cell adhesion and migration during the process of development (9).

## 2 Materials and methods

### 2.1 Patient information

The study included a 2-year-old male child and his parents as participants. The patient had previously undergone a prenatal ultrasound examination, revealing the absence of his left kidney. The participant hailed from the Ulanqab City area of the Inner Mongolia Autonomous Region in mainland China and belonged to the Han Chinese ethnicity. It is noteworthy that the patient had no familial background of kidney disease and was the firstborn of his parents, both of whom displayed typical phenotypes.

### 2.2 Whole exome sequencing and sanger sequencing

The participant and his parents’ peripheral blood samples were obtained using an EDTA anticoagulant tube for genomic DNA extraction (10). The QIAamp DNA Blood Mini Kit (Qigen, Hilden, Germany) was utilized for this purpose (10). Library preparation and exome capture were performed using the Human Comprehensive Exome Panel (Twist Bioscience, CA) (10). Subsequently, the captured libraries were sequenced on the MGISEQ-T7 sequencer (BGI, Shenzhen, China).

The raw data was subjected to quality assessment using FASTQC, followed by alignment of the clean reads to the reference genome (GRCh37/hg19) using BWA software. Subsequently, duplicate removal and base quality recalibration were performed, and SNP and Indel variants were identified using the GATK pipeline. The identified variants were annotated using ANNOVAR and filtered based on minor allele frequencies (MAFs) of less than 0.5% for dbSNP, 1000G, ExAC, and gnomAD databases (10).

In order to ascertain the existence of the variant, Sanger sequencing was conducted on the participant’s DNA extracted from peripheral blood.

### 2.3 Analysis of the deleterious effects of missense variants in the *TNXB* gene

The protein sequences of various species were obtained from NCBI, and subsequently subjected to multiple sequence alignment and conservation analysis using Jalview software (10).

### 2.4 3-D structure analysis of *TNXB*

The 3-D structure of the *TNXB* wild- and mute-type proteins were generated via homology modeling through MODELLER software by choosing AF-P24821-F1 protein as the template. The resulting structures were subjected to structural optimization by GROMACS version 2020.4 (http://manual.gromacs.org). The AMBER14SB force field and TIP3P water model were used for all simulations. The protein structures were visualized via VMD software (https://www.ks.uiuc.edu/Research/vmd) (10).

### 2.5 Molecular dynamics

GROMACS software package was used to simulate the protein molecular dynamics (11, 12). The protein uses the AMBER14sb force field. The protein was loaded into the GROMACS module, and hydrogen atoms and NaCl ions were added. Select TIP3P dominant water model and set periodic boundary conditions. The workflow of molecular dynamics simulation includes four steps: energy minimization, NVT equilibrium, NPT equilibrium and production dynamics simulation. Firstly, the protein heavy atoms were constrained to minimize the energy of water molecules by 10000 steps (including 5000 steps steepest descent method and 5000 steps conjugate gradient method); Then, maintaining the constraints, 50000 step NVT ensemble simulation was carried out for the whole system. The temperature was 298K, and the time step was 2fs; Then 50000 step NPT ensemble simulation was carried out for the whole system, the temperature was 298k, and the time step was 2fs; Finally, the molecular dynamics simulation of the system was carried out in the NPT ensemble for 100ns with a time step of 2fs. The relevant parameters were analyzed by the module of the GROMACS software package.

## 3 Results

### 3.1 Pathologic diagnosis

**Figure 1:**
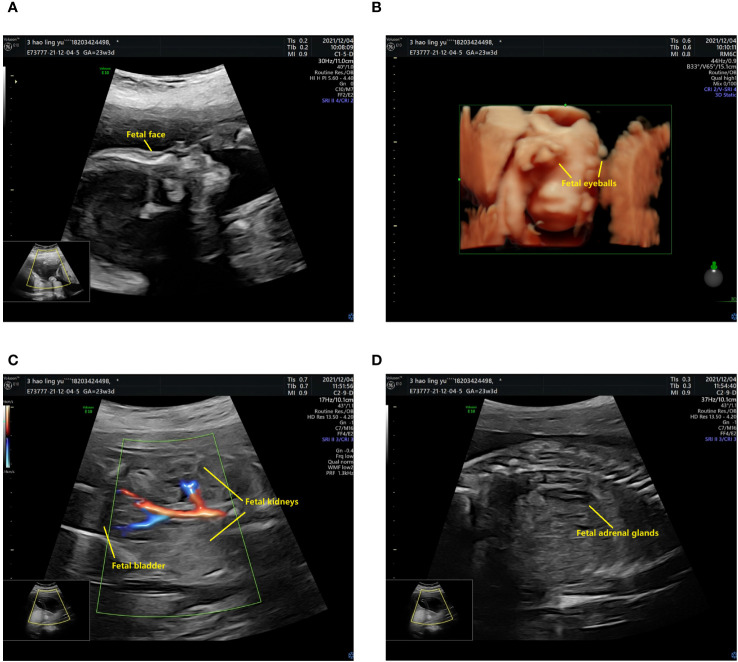
The prenatal ultrasound findings of the patient. **(A)** The fetal head and face exhibit several notable features. The skull is clearly discernible, with the midline of the brain appropriately aligned. The thalamus is visible on both sides, and the morphology of the cerebellar hemispheres appears normal without any evident abnormalities. Additionally, the cerebellar earthworms are visible, and there is no significant enlargement observed in the pool of the posterior cranial fossa. **(B)** The presence of bilateral eyeballs in the fetus is evident, and there is no apparent disruption in the continuity of the skin echoes of the upper lip. **(C)** The fetal abdomen and viscera exhibit visibility of the liver, stomach, left kidney, and bladder, with no apparent disruption in the echogenicity of the abdominal wall. The left kidney measures 33x14 mm in size, with a separated left renal pelvis measuring approximately 5 mm in width. There is no notable renal echo in the right renal region or pelvic cavity, and the right adrenal gland displays a “lying down” sign. **(D)** CDFI imaging reveals only the left kidney, while the fetal cord consists of two umbilical arteries.

**Figure 2:**
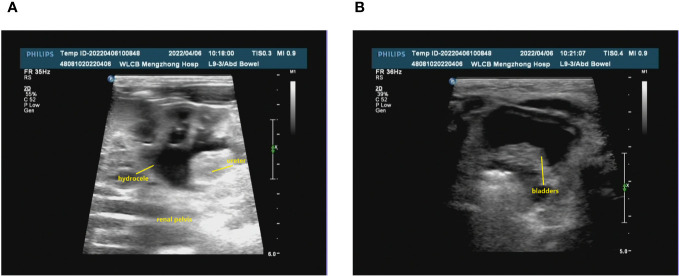
The renal ultrasound of the patient following childbirth. **(A)** Bilateral kidney assessment: The dimensions of the left kidney were approximately 48x28 mm, exhibiting homogeneous parenchymal echogenicity. No renal echogenicity was observed in the right renal region, and there were no discernible occupying lesions. The renal sinusoidal structure appeared clear, with no evident separation of the right collecting system. The left renal pelvis displayed widening, measuring approximately 9 mm in anteroposterior diameter. Additionally, the left ureter exhibited consistent dilation, reaching a maximum width of approximately 5 mm. **(B)** The bladder was adequately filled, featuring smooth walls and satisfactory intracapsular translucency.

The prenatal ultrasound findings indicated an intrauterine pregnancy with a solitary viable fetus and fetal right renal agenesis. The clinical gestational week was determined to be 23 weeks and 3 days, while the ultrasound gestational week was consistent with 22 weeks and 4 days. Additionally, the estimated weight of the fetus was approximately 522 ± 76 grams (**Figure 1**). Following childbirth, the postpartum ultrasound revealed left hydronephrosis and left ureteral dilatation, with the absence of the right kidney. No notable abnormalities were observed in the bladder (**Figure 2**).

### 3.2 Identification of TNXB mutation

**Figure 3:**
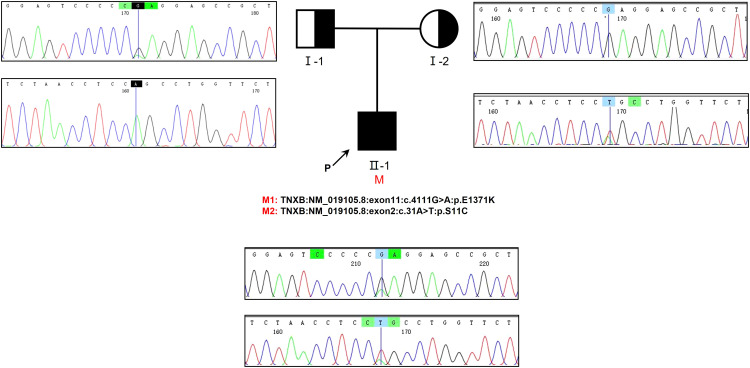
The examination of familial genealogy and the analysis of genetic sequencing in a patient originating from a Chinese family. The patient in question is indicated by the black arrow. Sanger sequencing was employed to confirm the genomic DNA of the patient and his parents. The presence of a mutation in the *TNXB* gene is denoted by the black vertical line. Within this family, we have identified two heterozygous missense variants that segregate, both of which were inherited in cis from one of the patient’s parents.

Genetic testing was conducted on the patient as well as his parents, yielding significant results. Specifically, the analysis revealed the presence of two compound heterozygous missense variants, namely c.4111G>A and c.31A>T, within the patient’s *TNXB* gene. The frequencies of the currently identified variants c.4111G>A and c.31A>T in the gnomAD East Asian population database are 0.000052 and 0.000231, respectively, and their documentation in the literature is currently absent. Importantly, it is worth noting that the patient’s parents did not exhibit any symptoms associated with kidney disease. Based on our findings, it can be inferred that the patient inherited the c.4111G>A variant from his father and the c.31A>T variant from his mother. Additionally, it is evident that both parents were heterozygous carriers for each of these two variants (**Figure 3**).

### 3.3 Analysis of abnormal structure of TNXB caused by mutation

**Figure 4:**
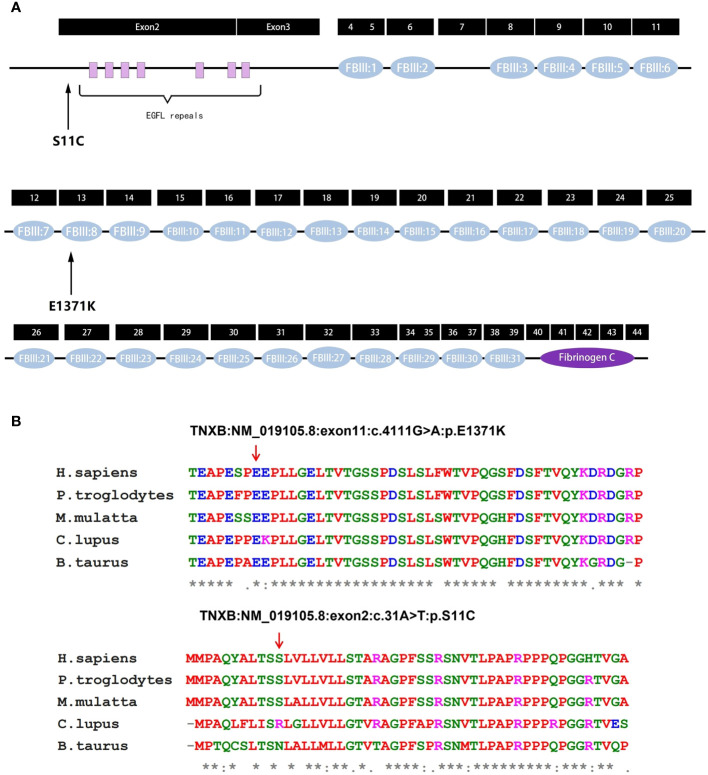
Analysis of *TNXB* mutations. **(A)** Exons and protein domains of *TNXB*. Filled boxes Exons of *TNXB* gene, filled ovals protein domains. The residue p.E1371K is situated within the eighth fibronectin type III (FBIII) structural domain of TNXB, while the residue p.S11C is situated within the N-terminal assembly structural domain preceding the EGFL-like repeat sequence. **(B)** Evolutionary conservation of amino acid residues altered by p.E1371K and p.S11C across different species. NCBI accession numbers are *Homo sapiens*: NP_061978.6; *Pan troglodytes*: XP_003311236.1; *Macaca mulatta*: XP_014983488.2; *Canis lupus familiaris*: XP_003431728.2; *Bos taurus*: NP_777128.1. The positions denoted by the red arrows correspond to glutamate position 1371 and serine position 11 within the *TNXB* protein. * indicates that the locus is highly conserved in the species. An "*" (asterisk) indicates positions which have a single, fully conserved residue. A ":" (colon) indicates conservation between groups of strongly similar properties. A "." (period) indicates conservation between groups of weakly similar properties.

**Figure 5:**
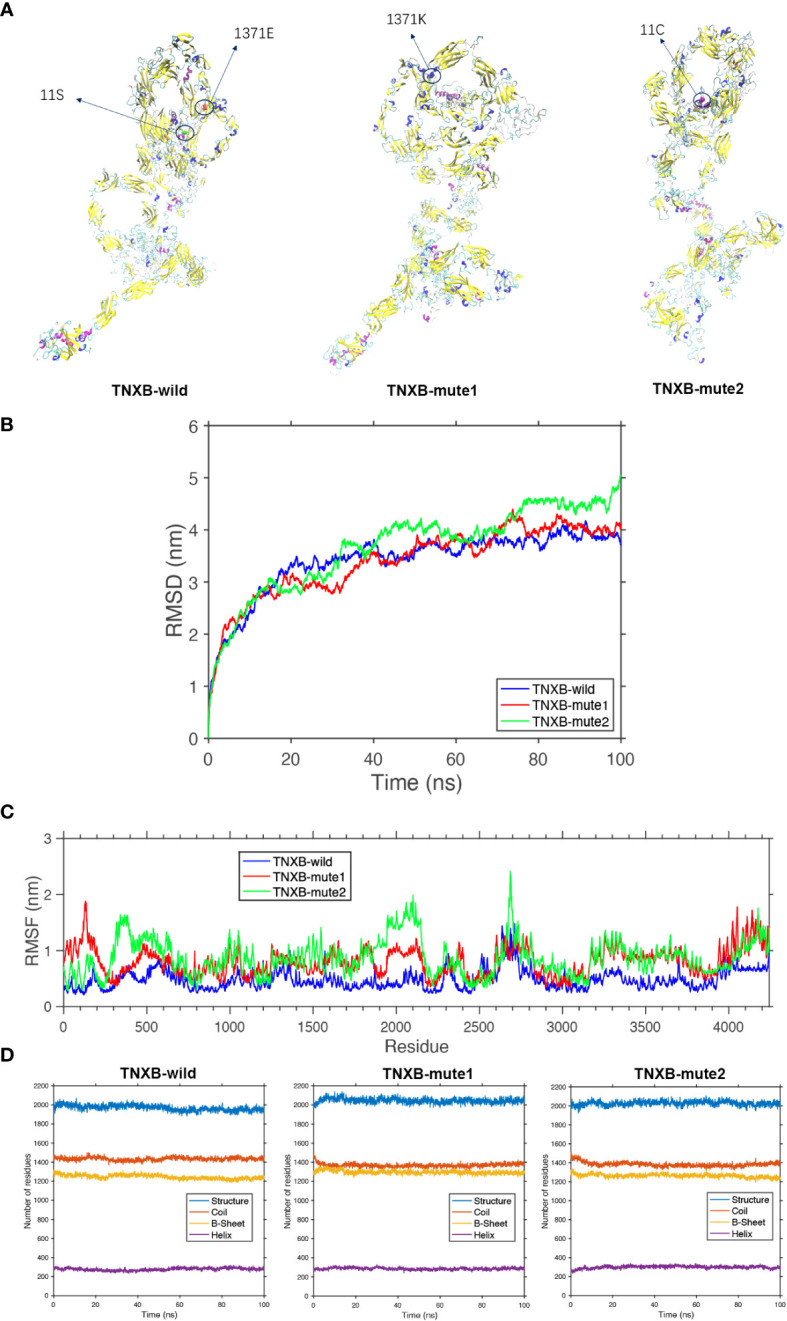
Protein molecular dynamics simulation of the *TNXB*. **(A)** The conformation of the *TNXB*-wild and -mute through molecular dynamics simulations at 100 ns. **(B)** RMSD of the *TNXB*-wild and -mute during 100 ns. **(C)** RMSF of the *TNXB*-wild and -mute during 100 ns. **(D)** The numbers of the secondary structures of the *TNXB*-wild and -mute during 100 ns.

The predicted location of p.E1371K is within the 8th fibronectin type III (FBIII) structural domains of *TNXB* (**Figure 4A**) (13). Upon modeling the entire p.E1371K mutation, it was observed that the mutation induces a modification in the secondary structure of FBIII 8 (**Figure 5A**). Similarly, p.S11C is situated in the N-terminal assembly structural domain of *TNXB* (**Figure 4A**) (13). A comprehensive modeling of the p.S11C mutation was conducted, revealing that the mutation disrupts the secondary structure of the N-terminal assembly structural domain (**Figure 5A**). Furthermore, examination of variant conservation predictions revealed that multiple species, along with patients exhibiting the E1371K substitution, possess the identical substitution (K) at position 1371. Likewise, at position 11, the amino acid serine (S) is substituted by arginine (R) in individuals with lupus and by asparagine (N) in those with taurus, both of which are polar amino acids akin to serine (**Figure 4B**).

### 3.4 Protein molecular dynamics simulation of the TNXB

The *TNXB*-wild has 1436 Coil structures, 1230 B-sheet structures, and 289 Helix structures. The *TNXB*-mute1(c.4111G>A) has 1395 Coil structures, 1305 B-sheet structures, and 300 Helix structures. Similarly, the *TNXB*-mute2(c.31A>T) has 1401 Coil structures, 1240 B-sheet structures, and 310 Helix structures. It is evident that the number of B-sheet and Coil structures has increased to varying degrees in both *TNXB*-mute1 and *TNXB*-mute2. The substitution of amino acid 1371 from E to K in *TNXB*-mute1 led to a transformation of the adjacent B-sheet structure into a Coil structure. Consequently, this mutation induced a structural alteration in this specific region of the protein chain, characterized by an augmented quantity of B-sheets and a diminished quantity of Coils when compared to *TNXB*-wild (**Figures 5A, D**). The alteration of amino acid 11 from S to C in *TNXB*-mute2 resulted in a transformation of the nearby Helix structure into a Coil structure. This mutation induced changes in the structure, specifically an increase in the number of B-sheets and a decrease in the number of Coils compared to the *TNXB*-wild. However, these changes were not as pronounced as those observed in *TNXB*-mute1 (**Figures 5A, D**). The root mean square deviation (RMSD) is employed to quantify the geometric disparity and demonstrates that *TNXB*-wild rapidly attains the equilibrium structure within 40ns, whereas *TNXB*-mute1 and *TNXB*-mute2 require a longer duration (~80ns) to achieve stability due to alterations in certain amino acids. This suggests that the mutation exerts a more pronounced influence on the protein’s conformational equilibrium. Furthermore, the fluctuation observed in *TNXB*-mute1 and *TNXB*-mute2 is slightly greater than that observed in *TNXB*-wild (**Figure 5B**). The root mean square fluctuation (RMSF) is utilized to quantify the distance (in nanometers) between each amino acid residue and its equilibrium site throughout the simulation. The magnitude of the peak signifies the extent of fluctuation exhibited by the respective amino acid residue. Upon comparing the fluctuations observed in *TNXB*-mute1, *TNXB*-mute2, and *TNXB*-wild, it becomes evident that mutations exert an influence on the fluctuation distances of certain amino acids within the protein chain, thereby significantly impacting the conformational fluctuations of the protein chain. Specifically, the *TNXB*-mute1 variant exhibited increased amino acid fluctuations in specific segments (100, 500, 1300-1400, 1900-2200, 2350-2400, and 3200-3800) as a result of the substitution of amino acid 1371 from E to K, while the *TNXB*-mute2 variant exhibited increased amino acid fluctuations in specific segments (300-500, 1900-2200, 2400, and 3200-3800) as a result of the substitution of amino acid 11 from S to C (**Figure 5C**).

## 4 Discussion

Chronic kidney disease (CKD) is a chronic and progressive ailment that significantly impacts the normal growth and development of children. The disease exhibits a subtle onset, with some cases eventually progressing to end-stage renal disease (ESRD), necessitating renal replacement therapy for life-sustaining purposes. The global prevalence of CKD ranges from 7% to 12%, while the prevalence of CKD in the G3 to G5 stages varies across different countries. Specifically, China reports a prevalence of 1.7%, Canada 5%, Australia 5.8%, and the United States 6.7% (14). In Europe, the prevalence increases from 2.3% in Germany and 2.4% in Finland to 4.0% in Spain and 5.2% in the United Kingdom. The epidemiological profile of CKD in low- and middle-income countries (LMICs) is limited due to the absence of community-based studies and the lack of consistent standardized assessment methods (14). Nevertheless, upon examining Southeast Asia, certain Latin American countries (such as Mexico), and sub-Saharan Africa, the prevalence of CKD consistently ranges from 10% to 16% (14). In 2014, the incidence of ESRD among children aged 0 to 14 years in Europe was 5.7 per million age-related population (pmarp), with a prevalence of 32.2 pmarp. Previous estimations indicated an incidence and prevalence of 8.3 pmarp and 58.0 pmarp, respectively, among children aged 0 to 19 years. These figures were lower than those observed in the United States for the age group of 0-21 years, which were 14.7 pmarp and 103.9 pmarp, respectively (14). There exist numerous etiological factors contributing to CKD in pediatric patients, with researchers positing that renal developmental anomalies are the predominant cause, constituting approximately 70% of cases. Among these anomalies, VUR represents a mild congenital abnormality within CAKUT phenotype. The CAKUT phenotype exhibits variability and has the potential to impact either the kidneys, the urinary tract, or both (15–17). CAKUT represent the prevailing etiology of end-stage renal disease in pediatric patients. Concurrently, additional manifestations of CAKUT, such as renal hypoplasia/hypoplasia, are frequently observed in conjunction with VUR. CAKUT can manifest either as an independent condition or as a component of a syndromic disorder, accompanied by extrarenal symptoms. Despite numerous genetic loci being identified in the last decade, the specific genes associated with VUR have yet to be determined. This study involved conducting genetic testing on a child presenting with hydronephrosis and possessing a solitary kidney, alongside their phenotypically unaffected parents. The results of our study indicate that the presence of compound heterozygous variants in the *TNXB* gene can be attributed to the development of hydronephrosis and single kidney manifestations in the specific child under investigation. These particular variants have been recently discovered and have not been documented in previous literature.

In our present study, the postnatal ultrasound of the child revealed the presence of left ureter dilatation accompanied by left hydronephrosis. The normal state of the ureter is one of non-dilation; however, in the presence of a malfunctioning anti-reflux mechanism within the ureter and bladder, urine reflux may occur, resulting in ureteral dilatation. Vesicoureteral reflux is characterized by the retrograde flow of urine from the bladder up into the ureter and ultimately the kidneys. Ordinarily, urine flows unidirectionally within the ureter. When obstruction prevents the passage of urine out of the kidneys, leading to its accumulation, renal swelling ensues, subsequently triggering the development of hydronephrosis. Hydronephrosis is not limited to a specific age group and can manifest at any stage of life. Hydronephrosis can be diagnosed in children during infancy, and in certain cases, prenatal ultrasound examinations may reveal the presence of hydronephrosis in fetuses. Hence, building upon prior research concerning the association between the *TNXB* gene and vesicoureteral reflux, we ascribe the clinical manifestation of hydronephrosis in this particular child to vesicoureteral reflux caused by mutations in the *TNXB* gene (5).

The precise contribution of *TNXB* mutations to the development of VUR remains uncertain. Nevertheless, it has been established that autosomal recessive *TNXB* gene mutations are linked to the manifestation of Ehlers-Danlos syndrome type III, characterized by joint hypermobility, diminished skin elasticity, and increased susceptibility to bruising. Chen et al. presented a case study involving an 8-year-old individual with a 46, XX karyotype, who was born with indeterminate genitalia, a bicornuate uterus, a solitary kidney, and grade III vesicoureteral reflux (18). Further investigation revealed that the individual had 21-hydroxylase deficiency. Upon examination at the age of 8, the individual exhibited a bifid uvula, a singular palmar crease on the left hand, normal skin, mild arachnodactyly, and hyperextensible joints, as evidenced by a Beighton score of 8 out of 9. On the other hand, the presence of heterozygous *TNXB* gene mutations leads to the manifestation of vesicoureteral reflux-8 (VUR8). Gbadegesin et al. documented the presence of radiologically confirmed vesicoureteral reflux and/or duplex collecting systems in a substantial 5-generation family (5). Among the individuals affected by these conditions, two mutation carriers exhibited indications of asymptomatic joint hypermobility upon examination. In the current investigation, we have discovered a previously unreported compound heterozygous missense variant of the *TNXB* gene within a limited familial lineage. Notably, the children within this lineage who displayed renal involvement did not manifest the typical joint hypermobility phenotype associated with Ehlers-Danlos syndrome type III. Intriguingly, the parents of the affected children were found to be heterozygous carriers of each of the two missense variants, yet neither exhibited any indications of vesicoureteral reflux. Consequently, we assert that the inheritance pattern of the *TNXB* gene is exceedingly intricate, and the precise molecular mechanisms involved warrant further exploration.

Tenascin-X (TN-X) is the largest member of the extracellular matrix glycoprotein family, which also includes Tenascin-C (TN-C), Tenascin-R (TN-R), and Tenascin-W (TN-W) (19). TN-X is unique in its significant structural function, as demonstrated by connective tissue disorders in both humans and mice when this glycoprotein is lost. The TNXB gene, encoding TN-X, was serendipitously discovered as an unknown (‘X’) gene located in the class III region of the HLA locus on human chromosome 6p21 (19). It was found to overlap with the gene coding for steroid 21-hydroxylase (CYP21A) in its 3’ portion. The discovery of TNXB was made possible due to this overlap with the human CYP21A2 gene. The genes encoding C4 and steroid 21-hydroxylase are in a single transcriptional orientation within a restricted region of the human class III major histocompatibility locus located at 6p21.3. However, the TNX gene, which overlaps the terminal exon of the CYP21 gene on the complementary DNA strand, exhibits the opposite transcriptional orientation. This intricate motif is replicated into two distinct motifs, labeled A and B, resulting in the following orientation: 5’-C4A-21A-TNXA-C4B-21B-TNXB-3’ (19). Chromosome walking experiments and sequencing work on the ‘X’ gene predicted a 450 kDa glycoprotein with five distinct structural domains (19). These domains include a signal peptide, a hydrophobic structural domain with four heptad repeats, an epidermal growth factor (EGF)-like structural domain with 18.5 repeats, a substantial fibronectin type III (FNIII) module, and a fibrinogen (FBG)-like globular domain at its C-terminus. This protein, known as Tenascin-X (TN-X), shares a modular structure with the prototypical TN-C glycoprotein and TN-R protein, which were discovered simultaneously. TN-X is the largest member of the Tenascin family, while TN-R and the newly discovered member TN-W are the smallest members (20). The human TN-W monomer has an apparent molecular weight of 160 kDa, while human TN-R has two major protein forms of 160 kDa and 180 kDa, and human TN-C has a monomer with molecular weights ranging from 190 kDa to 300 kDa (20). Alongside the cloning and characterization of the TNXB gene, TN-X was independently identified as a flexible glycoprotein associated with collagen fibers, initially known as flexin (21). *TNXB* is a member of a vast family of extracellular matrix proteins that are distinguished by the presence of an N-terminal assembly structural domain, EGF-like repeats, multiple fibronectin III structural domains, and a C-terminal fibronectin-like structural domain (5–9). The gene exhibits expression in the kidney and urinary tract throughout the developmental process, with particularly high expression during the crucial phase of ureterovesical bladder junction (UVJ) formation (5). However, the precise mechanism through which *TNXB* variation results in the occurrence of a solitary kidney remains to be fully understood. Based on prior research, it has been observed that fibroblast cell lines derived from individuals harboring the T3257I missense variant in *TNXB* display diminished cellular motility and decreased expression of phosphorylated adhesion spot kinase upon exposure to platelet-derived growth factor (PDGF). These findings imply a sustained and intensified cellular adhesion (5). PDGF is a fundamental protein that is sequestered within platelet alpha granules. It possesses a low molecular weight and functions as a pro-cytokinin. Its primary role is to induce cellular activation and proliferation, particularly in fibroblasts that are arrested in the G0/G1 phase (22). Renal interstitial fibroblasts, which establish a supportive framework alongside peritubular capillaries, are a specific cell population found within the renal interstitium (23). Two variants, p.E1371K and p.S11C, were observed in the linker region connecting the fibronectin domain with the EGF-like repeat region. Initially, it was hypothesized that the variant in the fibronectin domain primarily affects the motility of renal interstitial fibroblasts, while the variant in the EGF-like repeat region mainly affects the growth and differentiation of renal mesenchymal fibroblasts (5, 22). However, molecular dynamics simulation results revealed significant structural changes in the two TNXB mutant proteins, p.E1371K and p.S11C, compared to the wild-type protein. These changes were particularly prominent in the amino acid residues range of 100-500, 1900-2200, 2400, and 3200-3800. This disruption prevents the formation of the kidney structure scaffold, resulting in severe damage to kidney development. However, the reason behind unilateral renal impairment in the patients, rather than bilateral impairment, remains unclear. In the subsequent phase of our investigation, we intend to conduct a more comprehensive examination of cell motility in fibroblast cell lines harboring p.E1371K and p.S11C variants, employing scratch wound healing assays. The wound healing assay, also known as the scratch assay, is a commonly employed technique *in vitro* to evaluate the migratory potential of cell populations. This method relies on the observation that upon the creation of an artificial gap, or scratch, in a fully confluent monolayer of cells, the cells situated at the periphery of the scratch migrate towards the gap and form new intercellular connections, ultimately leading to the closure of the area. The experimental procedure encompassed the generation of ‘wounds’ in the monolayer of cells, followed by the acquisition of images at various time intervals to track the advancement of the ‘healing’ process. Subsequently, the collected data was subjected to analysis in order to determine the extent of closure in terms of percentage of the area, as well as to evaluate the rate of cell migration.

## 5 Conclusions

In summary, the identification of compound heterozygous variants c.4111G>A and c.31A>T in the TNXB gene in a afflicted child serves to broaden the range of variants observed in this gene. The confirmation of pathogenicity for these compound heterozygous variants was achieved through prenatal and postnatal ultrasound findings in the afflicted child. Consequently, the findings of this study enhance our comprehension of the molecular pathogenesis of VUR.

## Data availability statement

The datasets utilized in this study are available on ClinVar (https://www.ncbi.nlm.nih.gov/clinvar/RCV001281232.2/).

## Ethics statement

The studies involving humans were approved by Ethics Committee of Inner Mongolia Medical University. The studies were conducted in accordance with the local legislation and institutional requirements. Written informed consent for participation in this study was provided by the participants’ legal guardians/next of kin. Written informed consent was obtained from the individual(s), and minor(s)’ legal guardian/next of kin, for the publication of any potentially identifiable images or data included in this article.

## Author contributions

LL: Methodology, Conceptualization, Writing – original draft, Writing – review & editing. HW: Writing – review & editing. HM: Resources, Writing – review & editing. LF: Visualization, Writing – review & editing. JZ: Project administration, Funding acquisition, Resources, Writing – review & editing.
